# Local and system mechanisms for action execution and observation in parietal and premotor cortices

**DOI:** 10.1016/j.cub.2021.04.034

**Published:** 2021-07-12

**Authors:** Carolina G. Ferroni, Davide Albertini, Marco Lanzilotto, Alessandro Livi, Monica Maranesi, Luca Bonini

**Affiliations:** 1 Department of Medicine and Surgery, University of Parma, via Volturno 39, 43125 Parma, Italy; 2 Department of Psychology, University of Turin, via Verdi 10, 10124 Torino, Italy; 3 Department of Neuroscience, Washington University in St. Louis, St. Louis, MO 63110, USA

**Keywords:** action organization, mirror neuron, parietal cortex, premotor cortex, cell classes, pyramidal neurons

## Abstract

The action observation network (AON) includes a system of brain areas largely shared with action execution in both human and nonhuman primates. Yet temporal and tuning specificities of distinct areas and of physiologically identified neuronal classes in the encoding of self and others’ action remain unknown. We recorded the activity of 355 single units from three crucial nodes of the AON, the anterior intraparietal area (AIP), and premotor areas F5 and F6, while monkeys performed a Go/No-Go grasping task and observed an experimenter performing it. At the system level, during task execution, F6 displays a prevalence of suppressed neurons and signals whether an action has to be performed, whereas AIP and F5 share a prevalence of facilitated neurons and remarkable target selectivity; during task observation, F5 stands out for its unique prevalence of facilitated neurons and its stronger and earlier modulation than AIP and F6. By applying unsupervised clustering of spike waveforms, we found distinct cell classes unevenly distributed across areas, with different firing properties and carrying specific visuomotor signals. Broadly spiking neurons exhibited a balanced amount of facilitated and suppressed activity during action execution and observation, whereas narrower spiking neurons showed more mutually facilitated responses during the execution of one’s own and others’ action, particularly in areas AIP and F5. Our findings elucidate the time course of activity and firing properties of neurons in the AON during one’s own and others’ action, from the system level of anatomically distinct areas to the local level of physiologically distinct cell classes.

## Highlights

• F6 neurons show a prevalence of suppressed activity, encoding whether to act
• Area F5 and AIP share a prevalence of facilitated neurons and target selectivity
• Across-areas, waveform-based clustering distinguished three neuronal classes
• Narrow-spiking neurons exhibit mutual modulation during self and others’ action

## Teaser

A system of parietal and frontal areas in the primates’ brain is shared by action execution and observation. In this single-cell study, Ferroni et al. shed new light on the time course of system-level processing of executed and observed actions in the monkey and reveal differential contributions of waveform-based classes of neurons.

## Introduction

Action execution and observation recruit the same neural substrates in a wide set of brain regions in both human^1^, ^2^, ^3^ and nonhuman primates.^4^, ^5^, ^6^ Indeed, after the discovery of mirror neurons, a class of cells in the premotor area F5 of the macaque that become active during both the execution and observation of actions,^7^^,^^8^ similar neuronal properties have been found in a larger network of anatomically connected brain regions,^9^, ^10^, ^11^ which form the so-called action observation network (AON). The ventral premotor area F5 is thought to be the core of the AON and is certainly the most widely studied region.^4^^,^^12^ More recently, two other AON areas have attracted increasing interest: the anterior intraparietal area (AIP) and the pre-supplementary area F6. AIP plays a role in routing to F5 visual information regarding manipulative actions of other^13^, ^14^, ^15^ and area F6 hosts neurons that selectively encode actions and targets of self and others.^11^^,^^16^, ^17^, ^18^, ^19^ Despite these recent advances in our understanding of the AON, two critical questions remain unanswered.

First, what are the temporal and neuronal tuning specificities of the different areas of the AON? fMRI studies in humans^2^^,^^20^ and monkeys^9^^,^^21^ provide a system-level view of some areal specificities but cannot address their activation dynamics.

Second, how are self and other’s actions represented by different cell classes in the AON? The only available evidence comes from two previous studies demonstrating that a set of antidromically identified pyramidal-tract neurons in F5^22^ and F1^23^ exhibit mirror properties, often showing suppressed activity during action observation. A recent study provides a new unsupervised methodology to identify extracellularly recorded neuronal classes,^24^ but their possible functional specificities across and within different nodes of the AON remain unknown.

To address these issues, we extracellularly recorded neuronal activity from AIP, F5, and F6 in the AON using the same execution (EXE) and observation (OBS) tasks, and we extracted single neuron action potentials by applying a fully automated spike-sorting approach.^25^ Then, we compared single neuron and population codes among the three areas to obtain a functional fingerprint of the areal specificities in planning, execution, and observation of actions. Next, we pooled together all the recorded neurons and applied an unsupervised clustering of spike waveforms to identify distinct cell classes regardless of the area of origin. We found that cell classes (1) showed different properties in the execution and observation tasks, (2) were unevenly distributed across the investigated areas, and (3) made a substantial and differential contribution to areal functional specificities.

## Results

**Figure 1:**
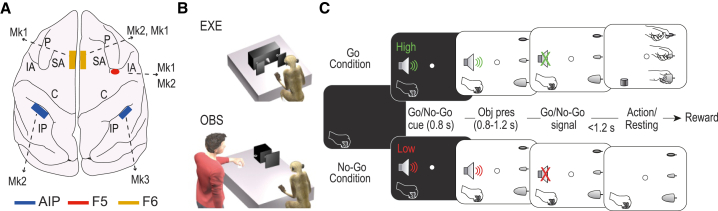
Recorded regions and behavioral task (A) Schematic reconstruction of the recorded regions in the three animals reported on Mk2’s brain. C, central sulcus; IA, inferior arcuate sulcus; IP, intraparietal sulcus; P, principal sulcus; SA, superior arcuate sulcus. (B) Behavioral setting for the execution (EXE) and observation (OBS) tasks, run in blocks (EXE first). (C) Temporal sequence of events of the Go/No-Go visuomotor task. The monkey starts with its hand in a fixed position. The onset of central fixation in the position where the object will be presented triggers a Go/No-Go auditory cue (high-/low-frequency sound, respectively). Following a variable delay after object presentation, the end of the sound (Go/No-Go signal) instructs the monkey to reach and grasp the visually presented object or to remain still until the end of the trial to obtain the reward. The different types of trials (Go/No-Go and object type) within EXE and OBS blocks were presented in a randomized order.

We isolated 436 units from three monkeys. All units with atypical features relative to a predefined set of criteria (STAR Methods) were excluded (n = 81, 18.6%), leading to a dataset of 355 well-isolated single neurons in three cortical areas (Figure 1A): AIP (n = 86), F5 (n = 106), and F6 (n = 163). During the recordings, monkeys performed an execution task (EXE; Figure 1B) and observed an experimenter performing the same task (OBS; Figure 1B). The temporal sequence of events was the same in both tasks (Figure 1C).

### Functional fingerprint of parietal and frontal areas during task execution and observation

**Figure 2:**
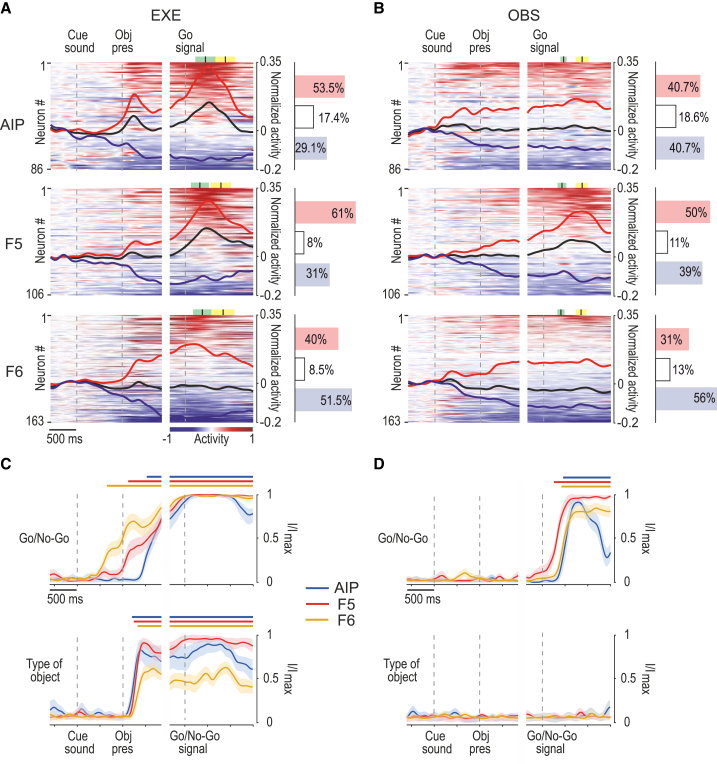
Functional fingerprint of parietal and frontal areas during task execution and observation (A) Heatmaps of all the recorded neurons in each area during EXE. Each line represents one cell (average activity of the responses to all the three objects). Cells are ordered (from top to bottom) based on the magnitude of their activity with respect to baseline (red, facilitated; blue, suppressed) in the interval between 300 ms before and 900 ms after the Go signal, independently for EXE and OBS. Black lines represent the averaged response of each population as a whole. The histograms on the right indicate the percentage of facilitated (red), suppressed (blue), and nonsignificant (white) neurons in each area (STAR Methods). Green and yellow marks represent average ± SD of movement onset and pulling onset, respectively. No-Go condition of EXE is shown in Figure S1A. (B) Heatmaps of all the neurons shown in (A) recorded during OBS. Data have been normalized together with EXE to facilitate comparisons. Note that the neurons have been ordered independently from (A) (see Figure S1E for OBS data plotted in the same order as in EXE). Other conventions as in (A). No-Go condition of OBS is shown in Figure S1B. (C) Mutual information on Go/No-Go trials (top) and type of object (bottom) during EXE decoded from neuronal population activity of each area during the task-unfolding period. Continuous colored bars above each plot indicate the period in which the mutual information is higher than 1/3 of its maximum theoretical value (STAR Methods). Mutual information about object during EXE is greater in both AIP and F5 relative to F6 (p < 0.05 for both comparisons; STAR Methods). Object decoding in No-Go condition of EXE is shown in Figure S1C. (D) Mutual information about Go/No-Go (top) and type of object (bottom) during OBS. Conventions as in (C). Object decoding in No-Go condition of OBS is shown Figure S1D.

To investigate the time course and functional specificities of neuronal processing during the tasks in the three areas, we first classified each neuron as facilitated (red), suppressed (blue), or nonsignificant (white) depending on its modulation during action execution (Figure 2A) and observation (Figure 2B) relative to baseline (STAR Methods).

During EXE (Figure 2A), in AIP and F5 we found a similar proportion of facilitated and suppressed neurons (AIP versus F5, χ^2^ = 0.04, p = 0.8354), with an overall prevalence of facilitated ones, and both areas differed from F6 where, instead, cells with suppressed response prevailed (F6 versus AIP, χ^2^ = 8.62, p = 0.0033; F6 versus F5, χ^2^ = 12.22, p = 0.0005). Facilitated neurons exhibited clearly measurable peaks of activity already in relation to the visual presentation of the object, first in AIP (median, +230 ms) and F5 (+240 ms) and later on in F6 (+350 ms, Mann-Whitney test; F6 versus AIP, Z = 2.91, p = 0.0036; F6 versus F5, Z = 2.20, p = 0.0276; STAR Methods). In contrast, relative to the Go-signal, the facilitated neurons’ peak of activity showed the opposite trend, occurring earlier in F6 (+230 ms) than in both F5 (+400 ms, Z = 2.78, p = 0.0054) and AIP (+390 ms, Z = 2.49, p = 0.0127), which in turn did not significantly differ from each other (Z = 0.19, p = 0.85).

To better investigate the time course of different signals across the studied areas, we performed a neural decoding analysis^26^ by training and testing a Poisson naive Bayes classifier to discriminate between Go and No-Go conditions based on the population activity of each area (STAR Methods). The results (Figure 2C) show that the mutual information distinguishing Go and No-Go trials became significant much earlier in area F6 (−280 ms from object presentation) than in F5 (+100 ms, z test on subsampling repetitions, Z = 2.60, p = 0.0092) and AIP (+440 ms, Z = 6.59, p = 4.3·10^11^), with F5 significantly preceding AIP (Z = 2.46, p = 0.0138). Conversely, mutual information about the type of target object emerges first in AIP (at 180 ms after object presentation), shortly thereafter in F5 (200 ms), and then in F6, significantly later (240 ms) compared to AIP (Z = 2.25, p = 0.024), but not to F5 (Z = 1.68, p = 0.092). The object-selective signal was both stronger and earlier in AIP and F5 relative to F6, where the mutual information about object type remained smaller than in the other two areas for the entire duration of the trial (Figure 2C, lower part). Interestingly, a stronger and earlier contribution of AIP in signaling the type of object is also made evident by an analysis of the neuronal population response during No-Go trials (Figure S1), supporting a predominantly visual nature of AIP object-related signal relative to F5 and F6.

Altogether, these findings highlight a greater similarity between the lateral convexity areas AIP and F5 than between either of those areas and F6, with the AIP-F5 circuit playing a major role in the processing of graspable objects and reaching-grasping actions by linking visual features of the target, encoded in AIP, with specific motor plans for grasping it, represented primarily in F5.^27^^,^^28^ Area F6 differs strongly from both AIP and F5 in terms of the timing and strength of its object- and action-related response, showing earlier and predominantly suppressed activity signaling whether a forthcoming action will be performed or withheld.

During OBS (Figure 2B), the overall modulation of both facilitated and suppressed neurons was smaller than during EXE in all the investigated areas. The number of facilitated and suppressed neurons was perfectly balanced in AIP, similarly to F5 (χ^2^ = 0.66, p = 0.4175), where facilitated neurons were only slightly more numerous; in contrast, in F6, suppressed neurons clearly prevailed, especially relative to F5 (F6 versus F5, χ^2^ = 10.31, p = 0.0013; F6 versus AIP, χ^2^ = 4.27, p = 0.0388). The proportion of nonsignificant cells slightly increased in OBS relative to EXE in all three areas; nonetheless, area F5 still exhibited a clear-cut modulation during the agent’s reaching-grasping action due to the prevalence of facilitated neurons, which exhibited a measurable peak of activity corresponding to the observation of object pulling onset. Instead, areas AIP and F6, despite hosting some single neurons with transiently facilitated activity during reaching-grasping observation (see heatmap in Figure 2B), did not show any phasic modulation of their population response.

By applying the neural decoding approach to OBS (Figure 2D), the classifier could detect significant mutual information discriminating between Go and No-Go trials only during the movement epoch, essentially revealing a robust signal related to action observation in all three areas. However, as compared to EXE (Figure 2C), we found no additional object or observed grip-type specificity during OBS. Significant mutual information about Go/No-Go raises earlier in F5 (+200 ms relative to the Go/No-Go signal) than in F6 (+360 ms, Z = 2.90, p = 0.0038) and AIP (+400 ms, Z = 3.11, p = 0.0019). Because neurons in different areas were not recorded simultaneously, hence being potentially subject to variation in the reaction time of the actor, we also repeated this analysis by aligning the activity of Go trials to reaching movement onset: the findings confirm the earlier activation of area F5 (−260 ms relative to movement onset) with respect to both AIP (−40 ms, Z = 3.55, p = 3.8·10^−4^) and F6 (0 ms, Z = 3.23, p = 0.0012).

These data lend strong support to the idea that, in the action observation network, area F5 does not necessarily need to be triggered by visual information about others’ actions coming from the parietal cortex^4^^,^^29^ but can also predictively represent upcoming actions of others^30^ with inherently generative capacities.^31^, ^32^, ^33^

### Identification and functional properties of cell classes based on extracellular spike waveforms

**Figure 3:**
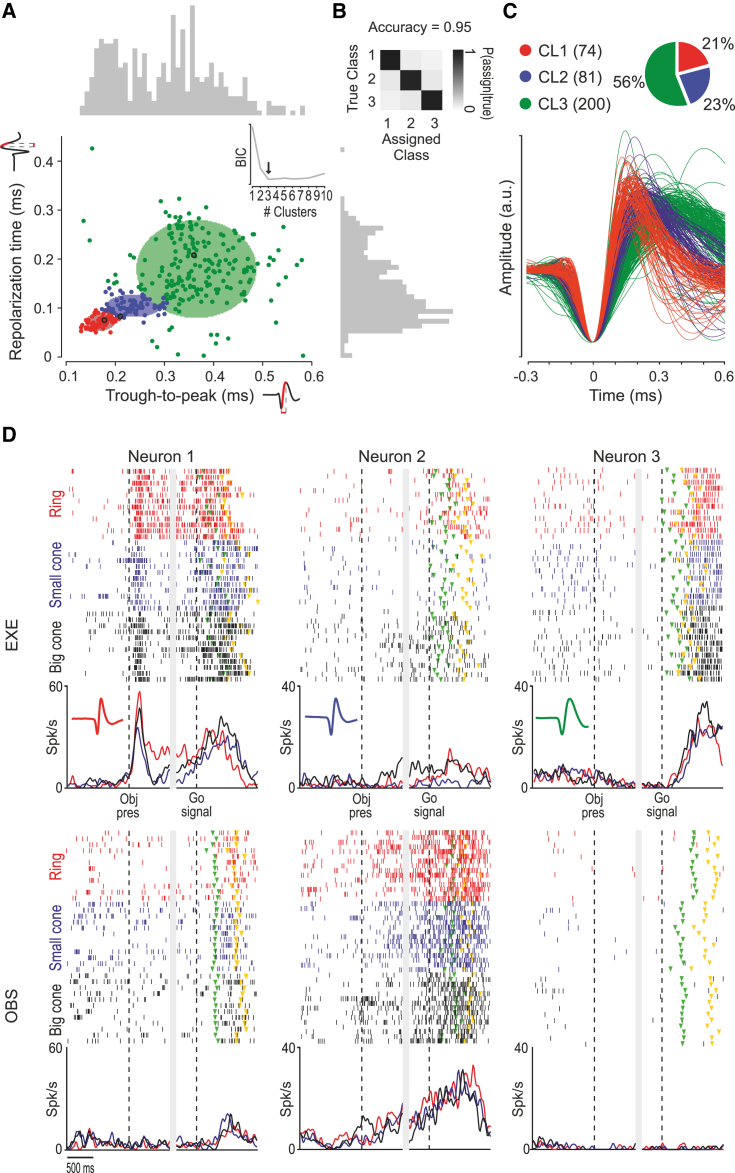
Identification and functional properties of cell classes based on extracellular spike waveforms (A) Projection of each spike waveform in the 2D space formed by trough-to-peak duration and repolarization time. Color codes identify the clusters (cell classes) resulting from the Gaussian mixture model applied with the number of components (n = 3) indicated by the Bayesian information criterion (BIC) shown in the inset (STAR Methods). The black dots in each cluster indicate the example neurons shown in (D). Colored ellipses indicate, for each cluster, the 2D confidence interval. Trough-to-peak values range from 0.13 ms to 0.58 ms, and repolarization time values range from 0.0025 ms to 0.43 ms. Average variability in trough-peak estimation is 3.1 μs (95^th^ percentile = 7.9 μs); average variability in repolarization time estimation is 14.8 μs (95^th^ percentile = 65.4 μs). See Figure S2 for clustering reliability within and across areas. Figure S3A shows alternative clustering results obtained using spiking and waveform features. (B) Separation among cell classes. For each of 10^4^ data points randomly generated from the fitted Gaussian mixture distribution, we compared the true class from which the point was drawn with the class to which it was assigned. The confusion matrix shows the classification results; accuracy is 0.95 and results from the mean of the three diagonal probabilities.^24^ (C) Number of neurons in each cell class (in color code) in the entire dataset and individual average spike waveforms belonging to each class. (D) Example neurons recorded in AIP, F5, and F6 (from Neurons 1 to 3; see black circles in A), belonging to each of the three classes (spike waveform is shown in the inset of each histogram; color code as in B). Activity is aligned (vertical dashed lines) on object presentation (Obj pres) and then (after the gap) on the Go signal, in both tasks. Each color refers to trials with one type of target object: a ring (red), a small cone (blue), and a big cone (black). Triangular markers indicate the movement onset (green) and object pulling onset (yellow).

Next, we wanted to investigate cell-class specificities of each of the areas described so far. To this end, we measured two parameters of spikes waveforms for all the neurons isolated in the three investigated areas, namely, trough-to-peak duration and repolarization time.^24^ The trough-to-peak duration defines the spike amplitude in terms of the interval between the global minimum of the spike shape and the following local maximum, whereas the repolarization time is the interval between the local maximum following the global minimum and the subsequent inflection point of the curve (Figure 3A).

To identify two-dimensional clusters with the available parameters and waveforms, we adopted an unsupervised clustering procedure (Gaussian mixture model; STAR Methods). A Bayesian information criterion (BIC) indicated the optimal number of Gaussian components (i.e., three waveform classes) in our dataset (Figure 3A, inset). The overall representation of the clustering results revealed three well-separated neuronal classes (Figure 3B) ranging from narrow spiking (class 1) to broad spiking (class 3) neurons, with a clear prevalence of broad spiking neurons (Figure 3C), in line with previous studies.^34^, ^35^, ^36^, ^37^

Representative examples of single neurons belonging to each of the three classes are shown in Figure 3D. Neuron 1 is an AIP cell belonging to class 1: during EXE, this neuron discharged vigorously during the presentation of the object and, subsequently, while it was being grasped, but it also fired during the experimenter’s grasping in OBS. Neuron 2 was recorded from area F5 and belongs to class 2: it discharged during the grasping of the ring and of the big cone in EXE and even more strongly during the experimenter’s grasping in OBS, but with no selectivity for the target object in this task. Finally, Neuron 3 is an F6 cell belonging to class 3: it reaches its peak of discharge during object pulling in EXE and shows no significant modulation during OBS.

**Figure 4:**
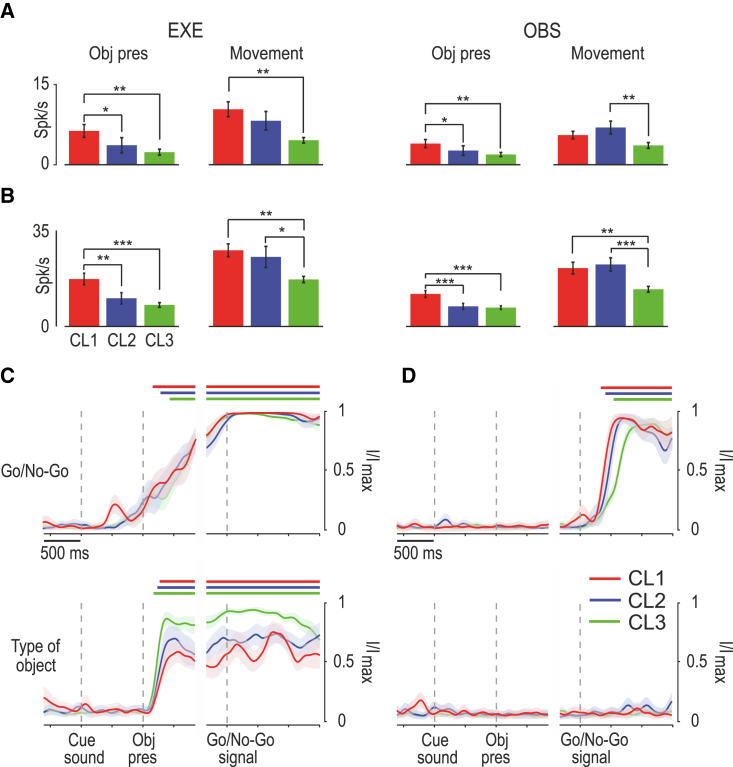
Cell-class response properties during EXE and OBS (A) Average net firing rates of facilitated neurons of cell class 1 (red), 2 (blue), and 3 (green) during object presentation (0.1 to 0.3 s relative to object presentations) and movement epoch (0.3 s before to 0.9 s after the Go-signal) in EXE (left) and OBS (right) tasks (one-way ANOVA with Newman-Keuls post hoc test). Cell class response is shown in Figures S4A and S4B. (B) Average peak of net firing rates of facilitated neurons of the three cell classes during object presentation (0.1 to 0.3 relative to object presentations) and movement epoch (0.3 s before to 0.9 s after the Go-signal) in EXE (left) and OBS (right). Conventions as in (A). ^∗^p < 0.05; ^∗∗^p < 0.01; ^∗∗∗^p < 0.001. Cell class response is shown in Figures S4A and S4B. (C) Mutual information on Go/No-Go trials (top) and type of object (bottom) during EXE decoded from neuronal population activity of each area during the task-unfolding period. Mutual information about object during EXE conveyed by neurons of Class 3 is greater than that of neurons of Class 1 (z test, p = 0.046) and 2 (p = 0.090). Conventions as in Figure 2C. (D) Mutual information about Go/No-Go (top) and type of object (bottom) during OBS. Conventions as in Figure 2C.

By comparing the firing properties of the cells in the three classes (regardless of the anatomical areas from which they were recorded), we reported several distinctive features. Although we generally found a greater number of facilitated than suppressed neurons (especially in class 1), their relative proportion did not differ significantly across classes in either EXE (Figure S4A) or OBS (Figure S4B); nonetheless, facilitated cells of classes 1 and 2 showed stronger average (Figure 4A) and peak (Figure 4B) activity during visual presentation of objects, and executed and observed actions, relative to cells of class 3. In turn, neurons of class 3 exhibit an earlier and remarkably stronger tuning to the object during EXE relative to the other two classes (Figures 4C and 4D). Thus, neurons with narrower spikes exhibit stronger visual and visuomotor responses, but they show a weaker object selectivity relative to broadly spiking neurons. In line with this latter observation, the firing statistics of the identified cell classes (Figure S4C) indicate that narrow spiking neurons exhibit a greater baseline firing rate, a shorter and more variable interspike interval (ISI), and a stronger tendency to fire in bursts than do broadly spiking neurons, which show a slower and more regular firing pattern.

### Functional specificities of cell classes in AIP, F5, and F6

**Figure 5:**
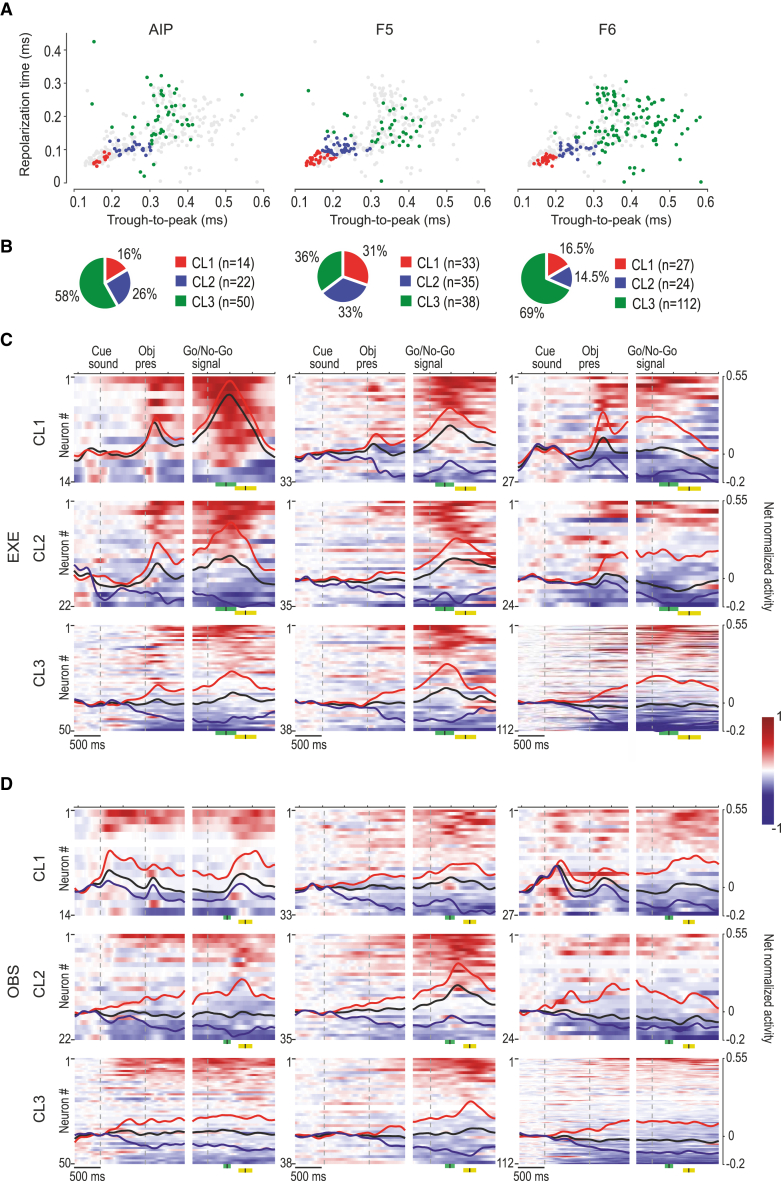
Functional specificities of cell classes in AIP, F5, and F6 (A) Projection of each spike waveform in the 2D space formed by trough-to-peak duration and repolarization time. Color codes identify the cell class to which each neuron of a given area has been attributed; gray dots in each plot correspond to the neurons that do not belong to the corresponding area. Other conventions as in Figure 3A. (B) Number of neurons of each cell class (in color code) in each area, expressed as a percentage of the total number of neurons recorded in each area. (C) Heatmaps and population response of all the recorded neurons recorded in each area during EXE, subdivided into the cell classes to which they belong. Conventions as in Figure 2A. Note that there are only 2 suppressed neurons in CL1 of AIP, so their average population line has not been plotted. Green and yellow marks represent average ± SD of movement onset and pull, respectively. See also Figure S5. (D) Heatmaps and population response of all the neurons recorded in each area during OBS, subdivided into the cell classes to which they belong. Conventions as in Figure 2C. See also Figure S6.

Based on the findings presented thus far, we then asked whether the identified cell classes (Figure 5A) contribute differently to the functional specificities of the three investigated areas. By comparing the overall distribution of neurons in the three classes (Figure 3C) with that obtained in each area (Figure 5B), we found no significant deviation in AIP (χ^2^ = 1.19, p = 0.55); furthermore, we found a greater proportion of neurons in the first two classes and a smaller number in class 3 in F5 (χ^2^ = 18.27, p = 0.0001), and the opposite trend in F6, which had a greater proportion of neurons in class 3 (χ^2^ = 10.57, p = 0.005). It is important to note that these results derive from a clustering applied to all the recorded neurons, pooled across areas, but we verified that they are extremely consistent and can be substantially reproduced even if clustering is performed within each area independently (Figure S2).

Next, we asked how neuronal classes contributed to the overall output signal of the three areas during EXE (Figure 5C). To this purpose, we applied a 3 × 3 × 3 repeated-measures ANOVA (within factor: epoch), with cell class and area as grouping factors, followed by a Newman-Keuls post hoc test where appropriate. The results (Figures 5C and S5) indicate that neurons of area F5 showed an overall stronger firing rate than those of both AIP and F6 (p < 0.001 for both comparisons), regardless of the cell class and, in particular, during the movement epoch relative to both baseline (p < 0.001) and object presentation (p < 0.001). Among cell classes, neurons of class 1 showed the overall highest firing rate, particularly in area F6; furthermore, they made the strongest contribution to object presentation (p < 0.005). These findings do not only depend on overall facilitated responses, but are also accounted for by the uneven distribution across cell classes and areas of suppressed neurons, which are particularly represented in F6 (Figure S5G).

The same analysis applied to OBS (Figures 5D and S6) confirmed the stronger activity of neurons in area F5 compared to those in AIP and F6 (p < 0.001 for both comparisons) regardless of the cell class and, in particular, during action observation relative to both baseline (p < 0.001) and object presentation epoch (p < 0.001), which in turn did not differ from each other (p = 0.2). Among cell classes, neurons of classes 1 and 2 showed greater firing rates during action observation relative to baseline and object presentation (p < 0.05); in particular, class 1 neurons of F6 exhibited a greater firing rate than neurons of classes 2 (p < 0.05) and 3 (p < 0.05) in the same area. The overall lower modulation of neuronal firing rate across epochs of OBS relative to EXE is likely due to a generally lower discharge of individual neurons during OBS than EXE and to the increased proportion of neurons (Figure S6F) showing unmodulated or suppressed response in this context. Suppressed neurons may play a role in balancing the overall motor output during action observation.^38^

### Mutual modulation of activity during action execution and observation: cell-class and areal specificities

As a final step, we asked whether and to what extent individual neurons’ modulation during the movement epoch of EXE and OBS jointly varied depending on area and cell class. Indeed, the only available evidence so far concerns antidromically identified pyramidal tract neurons of the ventral premotor^22^ and primary motor^39^ cortex, which often modulate their firing rate in an opposite manner during action execution and observation. Previous studies typically investigated this issue in individual areas and with an epoch-based approach,^22^^,^^39^, ^40^, ^41^, ^42^ which cannot be equally adapted to the firing properties of neurons in the various areas here investigated, where individual neurons’ activity has been tested with sliding t tests (STAR Methods).

**Figure 6:**
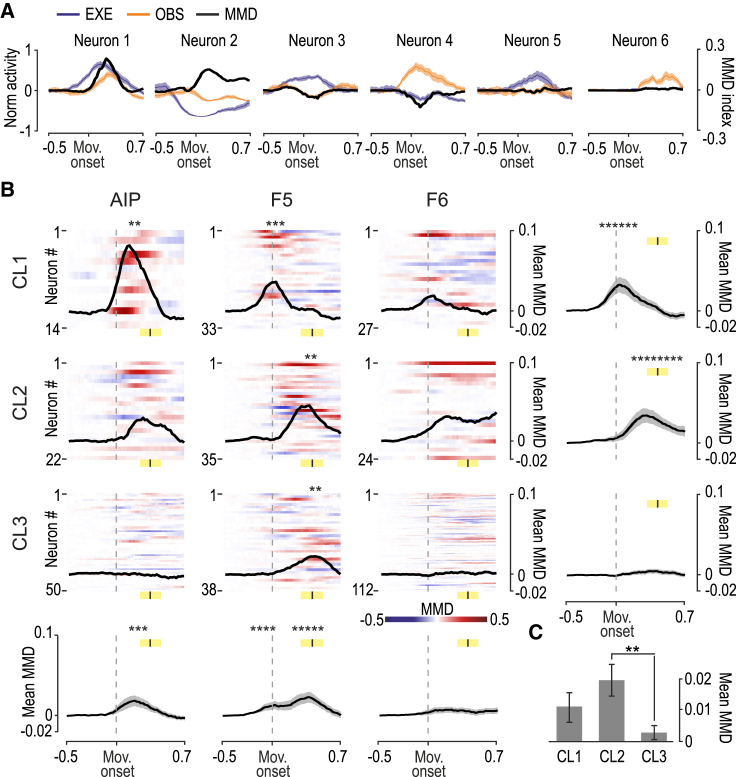
Mutual modulation of activity during action execution and observation: cell-class and areal specificities (A) Example of neurons showing a positive (Neurons 1 and 2), negative (Neurons 3 and 4), or flat (Neurons 5 and 6) MMD index during the movement epoch (aligned to the movement onset). Black curves represent the MMD time course, and colored trace represents the average ± SE net soft-normalized firing rates aligned to movement onset for EXE and OBS. (B) Heatmaps show the MMD time course for each neuron within areas and cell class (see color scale bar on the bottom right corner); black curves represent the average MMD values. Yellow marks represent average ± SD of object pulling time. Asterisks above each curve indicate sets of at least 5 consecutive time bins (200 ms in steps of 20 ms) of significantly increased MMD relative to the first 5 bins of the investigated period (one-tailed paired-sample t tests, p < 0.05). Each panel in the last row and column represents the time course of MMD index averaged across cell classes and areas, respectively; gray shadings represent SEs. Vertical black bars centered on the yellow shaded region represent average ± SD of pulling onset. The distribution and properties of neurons in each class depending on their modulation in EXE and OBS are shown in Figure S7. (C) Histograms show the mean MMD index (±SE) for each class. A 3 × 3 factorial ANOVA (factor: class and area) showed significant main effect of the factor cell class, and Newmann-Keuls post hoc tests revealed greater MMD of class 2 neurons relative to class 3 (p < 0.002).

Thus, to address this issue within cell classes and areas in our dataset, we devised an index to measure in a time-resolved manner the mutual modulation depth (MMD) of individual neurons’ discharge during EXE and OBS (STAR Methods). MMD values in the two tasks (Figure 6A) are closer to 1 the greater the positive (Neuron 1) or negative (Neuron 2) mutual modulation of the neuron’s activity in the two tasks, and are closer to −1 the greater the opposite positive-negative (Neuron 3) or negative-positive (Neuron 4) modulation of the neuron’s activity in EXE and OBS. MMD values are close to zero whenever a neuron’s discharge shows no modulation in any (Neurons 5 and 6) or both of the tasks. By looking at MMD changes of the different cell classes in each area (Figure 6B) during the task-unfolding period, we found increased MMD for cell class 1 and 2 following movement onset, particularly in AIP and F5, whereas neurons of cell class 3 did not show any relevant MMD change (with the exception of cells of class 3 in F5, which slightly increased their MMD later on, during object pulling). These findings indicate that neurons with narrow spikes exhibit stronger mutual modulation during action execution and observation.

## Discussion

In this study, we recorded single-neuron activity from three crucial nodes of the AON, the AIP and the premotor areas F5 and F6, during the execution and observation of reaching-grasping actions in a Go/No-Go paradigm. By leveraging the same tasks in all areas, we provided comparative evidence of temporal and neuronal tuning specificities at the system level and, at the same time, shed light on the cell-class coding principles that contribute to the AON functioning.

**Figure 7:**
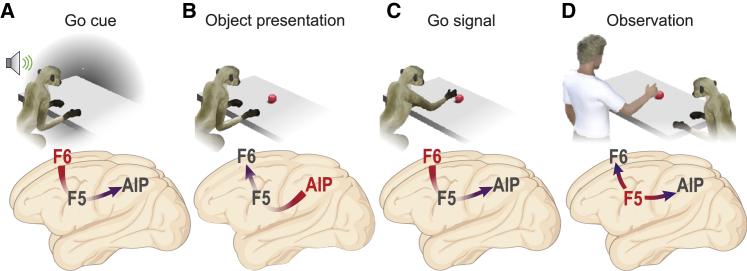
Schematic representation of the sequential contribution of AIP, F5, and F6 to tasks stages (A–C) Sequence of epochs of the execution task and time course of the activation (from red to blue) of the investigated areas in each epoch. (D) Experimenter’s movement epoch of the observation task.

During action execution (Figure 2A), more than half of AIP and F5 neurons exhibit a facilitated response, whereas in F6 the majority of neurons showed suppressed discharge. During task unfolding (Figure 7), area F6 neurons become active after cue sound onset, allowing us to decode whether an action will be performed earlier than in the other areas (Figure 2C); this information spread to F5 and finally to AIP (Figure 7A). When the target object is presented (Figure 7B), AIP generates an early and robust signal conveying object selectivity, closely coupled with that of F5: the object decoding accuracy obtained with the signal of these areas is followed by a lower and later object selective signal conveyed by area F6. This latter area reaches its peak of facilitated activity shortly after the Go signal, followed by that of F5 and AIP (Figure 7C), which are known to support proper execution of the visually guided grasping.^27^^,^^43^ These results favor a model in which F6 signals whether and when a forthcoming action has to be performed and receives feedback visuomotor information about graspable objects and ongoing actions from the AIP-F5 circuit.^28^^,^^44^

As illustrated in Figure 7D, and in line with existing evidence from anatomo-functional tracing studies,^10^^,^^11^^,^^41^ area F5 plays a key role in this network even during action observation. All areas showed weaker modulation in their overall activity during action observation (Figure 2B), both because of a generally lower discharge of individual neurons and a greater number of neurons showing suppressed discharge than during action execution. This applies particularly to AIP and F6. Indeed, F5 has a prevalence of facilitated neurons during both action observation and execution, and it is the only studied area with a cortico-spinal output^45^, ^46^, ^47^ and robust, direct connections to M1;^48^^,^^49^ furthermore, it shows a stronger and earlier modulation than do AIP and F6, consistent with recent neuronal population data emphasizing the representational similarity of executed and observed actions in F5.^42^^,^^50^ Importantly, control analyses of the temporal priority of F5 over the other two areas confirmed its capacity to actively generate a signal that not only anticipates the onset of another’s observed action^30^ but is also independent of the signal coming (260 ms later) from AIP or F6. Previous studies have demonstrated that F5 neurons can internally generate representations of external events even with limited^31^^,^^50^ or no^51^ visual information, and with very modest selectivity for the visual features of the stimuli.^33^ In line with previous work,^32^^,^^41^^,^^52^ we did not find object/grip-type selectivity in the observation task in any of the investigated areas. This finding could be due to the fact that monkeys were not paying attention to the details of the experimenter’s action because they were required to maintain fixation;^53^ previous studies with free-gazing monkeys did actually report object/grip selectivity in both parietal^13^ and premotor^54^ neurons recorded during observation of actions performed in the monkey’s peripersonal space. In our study, observing the action in a completely extrapersonal space^55^ may have further reduced object/grip selectivity. Thus, our findings support the idea that areas of the AON contribute to the temporal sequencing of motor events underlying others’ observed actions^50^ rather than their detailed perceptual analysis. Interestingly, in a predictive coding framework,^56^^,^^57^ the present findings suggest that among the tight reciprocal connections between F5, AIP, and F6,^10^^,^^11^^,^^41^^,^^58^ the projections carrying predictive signals from area F5 may have an overriding functional relevance in triggering neuronal activity at all levels of the network relative to feedforward information coming from visual areas, at least in highly predictable contexts. This model has recently received direct support from simultaneous recordings and chemogenetic manipulations of neuronal activity in the F5-to-F6 neural circuit, demonstrating that coordinated activity along this pathway has a causal role in social action monitoring.^59^

How do the key functional properties of the distinct nodes of the AON considered thus far map onto different cell classes? A null hypothesis would assume that distinct visuomotor functional properties are equally represented by different sets of neurons distinguished by their extracellular spike shape. The only attempt made so far for addressing this issue is constituted by studies that antidromically identified as pyramidal tract cells a set of F5^22^ and M1^23^ neurons and showed that they can exhibit mirror properties: interestingly, more than half of them suppressed their spontaneous activity during action observation. In the present study, we applied recently validated methods to perform an unbiased clustering of single-neuron waveforms, blind to the area of origin.^24^ In our dataset, we could distinguish three neuronal classes, varying in terms of their spike width from relatively narrow spiking (class 1 and 2) to broad spiking (class 3) neurons.^35^^,^^36^^,^^60^, ^61^, ^62^ By assessing cell-class responses in the execution and observation tasks, we found that narrow spiking neurons fired more strongly during baseline, object presentation, and action execution/observation and showed a greater tendency to fire in bursts relative to broad spiking neurons. These latter, in turn, exhibited slower and more regular firing patterns, with greater selectivity for the target during both object presentation and grasping execution relative to narrow spiking neurons. These differences among neuronal classes are consistent with those reported by earlier studies that have examined different tasks in other cortical areas.^63^, ^64^, ^65^

In terms of areal specificities, we found that F5 hosts a greater proportion of narrow spiking neurons, considering classes 1 and 2 together, whereas F6 exhibits the opposite trend, with a greater proportion of neurons belonging to class 3. Many previous studies suggested that neurons with narrow spikes correspond to putative interneurons,^66^, ^67^, ^68^, ^69^, ^70^ but a reliable association of class 1 (and at a certain extent, class 2) neurons with putative interneurons cannot be made as interneurons with broader spikes have been described as well.^71^ Furthermore, there are many issues that can influence spike width even among interneurons.^72^^,^^73^ Finally, there is evidence that in areas hosting big pyramidal cells, like F5,^74^ the bigger the pyramids the thinner the spike waveform:^39^ this may be a likely explanation for the prevalence of neurons in classes 1 and 2 in F5 relative to AIP and F6, which have smaller pyramidal cells as directly verified in histological slices of the brain regions investigated in the present study (Figure S3C). The fact that F5 has been recorded with daily inserted, movable linear probes^75^ may have further biased the sampling of bigger pyramidal cells with respect to the other two areas.

Interestingly, in all areas, especially AIP and F5, shared motor and sensory coding of one’s own and others’ action (as revealed by the MMD) is predominantly operated by more narrowly spiking neurons (classes 1 and 2), whereas broad spiking neurons (class 3) mostly encode either self- or other-related (unimodal) information. According to a previous hypothesis,^4^ cortico-cortical and cortico-striatal neurons could receive efference copies of motor actions encoded by cortico-spinal (pyramidal) neurons, supporting an evolutionarily ancient mechanism of sensorimotor remapping, which was previously demonstrated directly in songbirds.^76^ This mechanism has been shown to be optimized for shaping social responses and may also contribute to the previously observed overall suppression of discharge of pyramidal (especially corticospinal) neurons during action observation.

In summary, the present findings shed light on the temporal and network-level organization of self and others’ action in three of the recently recognized nodes of the AON in the monkeys. Although solely based on our results we cannot conclusively determine the correspondence between physiologically identified neuronal classes and their histological nature (e.g., pyramidal cell versus inhibitory interneurons), our findings suggest that visuomotor properties may be unevenly represented by distinct cell classes, possibly including inhibitory interneurons. However, cell-specific causal manipulation studies with optogenetic or neuropharmacological approaches^77^ are needed to investigate the possible correspondence between functional and morphologically identified cell classes, whose elucidation would considerably advance our understanding of the mechanisms underlying the wide range of perceptual and socio-cognitive functions implemented by the cortical motor system.

## STAR★Methods

### Key resources table

**Table:** 

REAGENT or RESOURCE	SOURCE	IDENTIFIER
**Experimental models: organisms/strains**		

Macaca mulatta	R. C. Hartelust	O Box 2170, Tilburg 5001 CD the Netherlands; Email: info@hartelust.net

**Software and algorithms**		

MATLAB	Mathworks	RRID: SCR_001622
LabView	National Instruments	RRID: SCR_014325
OmniPlex Software	Plexon	RRID: SCR_014803

### Resource availability

#### Lead contact

Further information and requests for resources and reagents should be directed to and will be fulfilled by the Lead Contact, Luca Bonini (luca.bonini@unipr.it).

#### Materials availability

Rhesus macaques used in this study were provided by R.C. Hartelust.

#### Data and code availability

The data and code supporting the current study are available upon request to the lead contact.

### Experimental model and subject details

#### Macaque monkeys

Experiments were performed on three purpose-bred, socially housed adult macaques, Mk1 (*M. nemestrina,* male, 9 kg), Mk2 (*M. mulatta,* male, 7 Kg) and Mk3 (*M. mulatta,* female, 4 Kg). Neuronal activity was recorded from two different monkeys per area (Figure 1A). Before recordings, the monkeys were habituated to sitting in a primate chair and interacting with the experimenters. Then, they were trained to perform an execution (EXE) and an observation (OBS) task,^51^ as described below. When the training was completed, a head fixation system and different types of probes were implanted (during distinct surgeries) as previously described elsewhere.^75^^,^^78^^,^^79^ All surgical procedures were carried out under general anesthesia (ketamine hydrochloride, 5 mg/kg intramuscularly [i.m.] and medetomidine hydrochloride, 0.1 mg/kg, i.m), followed by postsurgical pain medications. The experimental protocols complied with the European law on the humane care and use of laboratory animals (Directive 2010/63/EU), were authorized by the Italian Ministry of Health (D.M. 294/2012-C, 11/12/2012 and 48/2016-PR, 20/01/2016), and were approved by the Veterinarian Animal Care and Use Committee of the University of Parma (Prot. 78/12, 17/07/2012 and Prot. 91/OPBA/2015).

### Method details

#### Apparatus and behavioral paradigm

The apparatus for the visuomotor (EXE) and observation (OBS) tasks (Figure 1B) is described in details in a previous study.^51^ Briefly, during EXE, the monkey was seated on a primate chair in front of a box, divided horizontally into two sectors by a half-mirror where a spot of light (fixation point) was projected in the exact position of the center of mass of the not-yet-visible target object. The objects (a ring, a small cone, and a big cone) were presented randomly, one at a time, within reach of the monkey’s hand starting position. The objects afforded three different grip types: hook grip (ring), precision grip (small cone) and whole-hand prehension (big cone). The task included two basic conditions, Go and No-Go, and each trial was preceded by a variable (from 1 to 1.5 s) intertrial period.

In the Go condition the fixation point was presented, and the monkey was required to start fixating on it within 1.2 s. Fixation onset resulted in the presentation of a cue sound (high tone, 1200 Hz), which instructed the monkey to grasp the subsequently presented object (Go cue). After 0.8 s, one of the objects became visible. Then, after a variable time lag (0.8–1.2 s), the sound ceased (Go signal), and the monkey had to reach, grasp and pull (for 0.8 s) the object within 1.2 s to receive a fixed amount of juice reward (automatically delivered).

In the No-Go condition the sequence of task events was the same as in the Go condition, but a different cue sound (low tone, 300 Hz) instructed the monkey to remain still and fixate on the object for 1.2 s after the end of the sound in order to receive the reward.

The same sequence of events described for EXE also applied to OBS, in which an experimenter performed the task in the monkey’s extrapersonal space, seen by the monkey from a 90° visual perspective.^32^

Contact-sensitive devices (Crist Instruments) were used to detect when the monkey (grounded) touched the metal surface of the starting position or one of the target objects. To signal the onset and tonic phase of object pulling, an additional device was connected to the switch located behind each object. Custom-made LabView-based software was used to monitor the monkey’s performance and to control the presentation of auditory and visual cues.^32^ Eye position was monitored at 50 Hz with a camera-based eye tracking system and the monkey was required to maintain its gaze on the fixation point (with a tolerance radius of 5°) throughout the task. If the monkey broke fixation, made an incorrect movement or did not respect the task’s temporal constraints, no reward was delivered and the incorrectly performed trials were put back in the randomized list to be subsequently repeated. We collected at least 10 correctly performed trials for each condition.

#### Recording techniques

Neuronal recordings were performed by means of multielectrode linear silicon probes in different single-shaft^75^^,^^80^ or 3D^79^ configurations, implanted chronically in AIP^41^ and F6^40^ and acutely in F5,^51^ based on MRI reconstruction of the target brain regions. The analog signal from all the recording electrodes was simultaneously amplified and sampled either at 30 kHz with an OpenEphys system (http://open-ephys.org/) or at 40 kHz with an Omniplex system (Plexon).

All formal signal analyses were performed offline. Spike sorting was performed with fully automated software, MountainSort^25^ using −3.0 SDs of the signal-to-noise ratio of each channel as the threshold for detecting units. To discriminate single- from multi-units, we used the noise overlap parameter. This parameter, ranging between 0 and 1, estimates the fraction of “noise events” in a waveform cluster, i.e., above-threshold events not associated with well-isolated clusters. In most of the recording sessions, the noise overlap distribution is bimodal, with putative single-units associated with values below ∼0.1 and putative multi-units with values above ∼0.3. Thus, we considered as well-isolated single units only those with noise overlap values lower than 0.1. We then automatically inspected all waveforms of all isolated units and retained, for each unit, only those waveforms that did not exceed ± 3 SD from the average waveform in all data points (approximately 10% of the waveforms in each unit were removed with this procedure), to reduce the random variability and improve the accuracy in the extraction of spike shape parameters. Single unit isolation was further verified using standard criteria (ISI distribution, refractory period > 1 ms, and absence of cross-correlated firing with time-lag of ≈ 0 relative to other isolated units, to avoid oversampling).

To obtain the average waveform for each individual unit we randomly selected 1,000 of the filtered signal’s spikes in a window of 2.5 ms centered on the spikes’ absolute minimum. Each waveform was spline interpolated in order to achieve 1000 points in the 2.5-ms window, regardless of the original sampling rate, and realigned to the absolute minimum. This procedure produced the average waveform for all units. Then, we obtained the final dataset by excluding all units with 1) less than 1000 spikes (n = 15); 2) very noisy waveforms (multipeak, e.g., multiple local maxima between the main trough and the subsequent peak) (n = 35); 3) a main trough amplitude smaller than the subsequent peak or a peak before the trough greater than 20% the trough depth amplitude (n = 31), because they likely belong to axon fibers.^70^^,^^81^ The final dataset included 355 single neurons fulfilling all these criteria.

### Quantification and statistical analysis

#### Clustering of single-neuron waveforms

To cluster neurons, we first explored the possibility to use a combination of waveform parameters and firing features, but the results (Figure S3A) did not outperform those obtained with the two most widely established waveform parameters, namely, trough-to-peak duration^35^^,^^36^ and repolarization time.^24^ The trough-to-peak duration is the interval between the global minimum of the curve and the subsequent local maximum. Repolarization time is the interval between the late positive peak and the subsequent inflection point (where the second derivative equals zero); although it does not clearly correspond to the actual full repolarization of the cell membrane post-spike, it is a reliable predictor of this parameter.

Then, to identify clusters of waveforms based on these two parameters, we followed a recently described procedure^24^ in which the two-dimensional data points are fitted with a Gaussian mixture distribution (MATLAB function: fitgmdist). The procedure optimizes the likelihood Gaussian mixture model using the iterative Expectation-Maximization (EM) algorithm. Each iteration implies two steps: first, EM algorithm estimates posterior probabilities of each data point given the current set of component means, covariance matrices and mixing proportions (E step); then, using these probabilities as weights, it estimates new component means, covariance matrices and mixing proportions (M step) and evaluates the log-likelihood with these new parameters’ estimates. These steps are repeated until convergence or for a maximum of 100 iterations. To initialize the EM algorithm, we used k-means++ algorithm: 500 different replicates were run with different initializations and the model with the largest log-likelihood was adopted. For all the replicates, in order to reduce the number of free parameters, we imposed the covariance matrix of each component to be diagonal because even if trough-to-peak duration and repolarization time are generally correlated, this is not the case within individual clusters. We repeated this procedure by fitting the data with a different number of clusters (from 1 to 10), taking as the number of clusters the one that minimize the Bayesian Information Criteria (BIC, Figure 3A). We obtained three clusters (cell classes) with a variable number of neurons attributed by hard assignment, that is, by assigning each neuron to the cluster associated with the highest posterior probability. For visualization purposes, 68% confidence ellipses, i.e., the bivariate analog of the standard error, were shown for each cluster.^82^ Previous studies adopted an additional outlier removal procedure, which led to the exclusion of approximately 11% of the neurons;^24^ this procedure would have had a similar impact on our dataset, with 7% of the neurons excluded, more than 68% of them belonging to class 3, which includes the greatest number of neurons. Because in this study one of the main goals was to provide a comprehensive comparative picture of areal specificities, we decided not to remove otherwise fully valid physiological data by adding further exclusion criteria to those described above.

In order to look for additional support to the subdivision of neurons into functional classes and, more specifically, to further evaluate the possibility to functionally characterize some narrow spiking neurons as inhibitory interneurons, we applied cross-correlation analysis^83^^,^^84^ but the results did not provide sufficiently robust evidence to reach a sound conclusion on this issue (Figure S3B).

#### Population analyses

For each neuron, we first computed its baseline firing rate (corresponding to the 500-ms time interval preceding cue-sound presentation) for EXE and OBS (objects and trials averaged), separately. We then computed the net normalized activity of each neuron. First, we subtracted its baseline activity in a given condition from the firing rate of each bin; then, we soft-normalized the resulting net activity vector by dividing each data point by the absolute maximum across all conditions + 5 spk/s (this latter constant factor reduces the overall net normalized activity of neurons a with very low firing rate). The resulting net normalized activities (ranging theoretically between −1 and 1) were used to produce the heat-maps in order to show individual neurons’ firing rate in a comparable form during EXE and OBS task-unfolding periods.

Neurons were classified as facilitated or suppressed depending on the sign of the average modulation they showed during the movement period (action execution or observation in the time interval ranging from −300 ms before to 900 ms after the Go signal). To test whether the modulation of facilitated (red lines in Figures 2 and 5) and suppressed (blue lines in Figures 2 and 5) neurons was statistically significant, we compared their baseline activity with each bin of the movement period (one-tailed sliding t test, window = 200 ms, step = 20 ms, p < 0.05, uncorrected) in the −300/+900-ms interval around the Go signal during the entire movement period of EXE and OBS. We considered significantly facilitated or suppressed all those neurons with at least five consecutive significant bins, whereas neurons that did not meet this criterion were classified as non-significantly modulated. Note that this constitutes a very permissive statistical criterion relative to conventional epoch-based approaches.^32^ This choice was motivated by the fact that we did not want to study very restrictive and specific functional categories of neurons, but rather to include all the available cells and provide an (as much as possible) unbiased comparison of the three studied areas. Because they are known to possess different firing/temporal pattern of activity^40^ conventional epoch-based statistics would have strongly biased the results of the comparisons among areas.

The peak of activity times of facilitated neurons were calculated in the 100/500-ms time interval after object presentation and in 0/600-ms time interval after the Go-signal.

#### Decoding analyses

To compare how information about task parameters was represented in different areas, we employed the Neural Decoding Toolbox^26^ used in our previous studies.^14^^,^^18^^,^^41^ Specifically, we assessed the decoding accuracy of a Poisson naive Bayes classifier trained and tested to classify different variables, that is, Go/No-Go or type of object (Figures 2 and S1).

Regardless of the decoded variable, for each neuron, data were first converted from raster format into binned format. Specifically, we created binned data that contained the average firing rate in 200-ms bins sampled at 20-ms intervals for each trial (data point). We obtained a population of binned data characterized by a number of data points corresponding to the number of trials per conditions (i.e., 30 × 2 = 60 data-points for Go/No-Go decoding; 10 × 3 = 30 data-points for object decoding) in an N-dimensional space (where N is the total number of neurons considered for each analysis). Next, we randomly grouped all the available data points into a number of splits corresponding to the number of data points per condition, with each split containing a “pseudo-population,” that is, a population of neurons that could be partially recorded separately but treated as if they were recorded simultaneously. Before sending the data to the classifier, we pre-selected those features (neurons) that showed a difference between conditions with p < 0.5. Subsequently, the classifier was trained using all but one of the splits of the data and then tested on the remaining one. This procedure was repeated as many times as the number of splits (i.e., 30 in the case of Go/No-Go decoding, 10 in the case of object decoding), leaving out a different test split each time.

As a measure of the performance of the classification, we used the mutual information (MI^85^), defined as the reduction of uncertainty (or gain of information) about the current condition achieved by knowing the neuronal response. The greater the amount of information carried by the population, the smaller the uncertainty regarding the current condition. When the probability of presenting each of *K* different conditions is equal, MI can reach a theoretical maximum of *log*_*2*_*K* (i.e., 1 for Go/No-Go decoding and 1.585 for object decoding); we used these values to normalize MI corresponding curves in Figures 2 and S1. Because, on average, the higher the number of neurons used in the decoding, the higher the performance of the classifier, we performed a number-matching procedure to make the results of different areas comparable. To this end, we performed the decoding analysis on randomly selected sets of 65 neurons from each area (with replacement), corresponding to 3/4 of the neurons in AIP (n = 86), which is the area with the lowest number of neurons. We repeated this procedure 50 times, averaging each iteration across 10 runs with different data in the training and test splits from the same set of neurons and smoothing it with a 40 ms Gaussian kernel, to increase the robustness of the results. Finally, we computed the mean and the standard deviation (shading in Figure 2) of the resulting distribution.

To assess statistically when each area starts to convey a given type of information (i.e., Go/No-Go or object/grip type), we calculated for each iteration of the procedure described above the time point where the mutual information exceeds 1/3 of its maximum theoretical value. This calculation was repeated with all iterations and the standard deviation of the resulting time point distribution (multiplied by 65/*N*_*area*_ in order to consider the different subsample size with respect to the reference population) was taken as standard error. We compared the mean onset among areas by performing multiple two-tailed two-sample z-tests (p values uncorrected). We also compared how information about task parameters was represented among cell classes (Figures 4C and 4D). Since the investigated areas differently encode information about task events (Figures 2C and 2D), for each cell class we randomly sampled (with replacement) pseudo-populations including a fixed number (n = 20) of neurons of that class from each area. Decoding was performed on these 3 pseudo-populations (n = 60), and this procedure was repeated 50 times, averaging each iteration across 10 runs. Average mutual information curves and their significance were obtained as described above.

To assess the difference in mutual information about object type across areas (Figure 2C) and cell classes (Figure 4C), we used the same procedure described above on the average mutual information in the 200/700 ms interval after object presentation.

#### Index of mutual modulation depth

For the purpose of comparing the dynamic (positive or negative) modulation of single-neuron discharge in corresponding time bins of EXE and OBS, we created an index quantifying the mutual modulation depth (MMD). For each neuron, in the interval −500/700 ms relative to the movement onset, we calculated the net soft-normalized activity (as described above) separately for EXE and OBS, and we smoothed it with 200-ms (centered at intermediate values) bins advanced in steps of 20 ms. The MMD index was then computed for each neuron as the product of EXE and OBS activity values, as follows:

$$MMD_{n}\left(t\right)=EXE_{n}\left(t\right)\cdot OBS_{n}\left(t\right)$$

where *EXE*_*n*_*(t)* and *OBS*_*n*_*(t)* represent the net (500 ms prior to the Go signal) soft-normalized activity of neuron *n* during time bin *t* of EXE and OBS task, respectively. Neurons showing a similar discharge profile in both EXE and OBS (regardless of whether the neuron was jointly facilitated or suppressed) showed positive MMD values: the closer to 1 (theoretical value), the greater the (positive or negative) discharge modulation (Figure 6A, Neuron 1 and 2). In contrast, neurons showing large but opposite modulation in the two tasks (facilitated/suppressed or vice versa), showed negative MMD values: the closer to −1 (theoretical value), the greater the EXE and OBS opposite modulation (Figure 6A, Neuron 3 and 4). If in one condition the neuron does not modulate its discharge, the index tends to 0 regardless of the neuron’s modulation in the other condition (Figure 6A, Neuron 5 and 6).

To assess possible significant changes in overall MMD values during the movement epoch of specific neuronal subpopulations, we compared bin-by-bin MMD values with a fixed value corresponding to the average of the first 5 bins (300 ms of activity) of each plot (one-tailed paired sample t test, p < 0.01). We considered significant only series of at least five consecutive bins (black asterisks at the top of each plot of Figure 6B).

## Supplemental information

Document S1. Figures S1–S7

Document S2. Article plus supplemental information
