# Supplementary material for: Local and system mechanisms for action execution and observation in parietal and premotor cortices
Source: Curr Biol. 2021 Jul 12;31(13):2819–2830.e4. doi: 10.1016/j.cub.2021.04.034 (PMC8279740; doi:10.1016/j.cub.2021.04.034)
Supplement: Document S1. Figures S1–S7 [file mmc1.pdf]

Current Biology, Volume *31*

Supplemental Information

Local and system mechanisms for action execution

and observation in parietal and premotor cortices

Carolina G. Ferroni, Davide Albertini, Marco Lanzilotto, Alessandro Livi, Monica Maranesi, and Luca Bonini

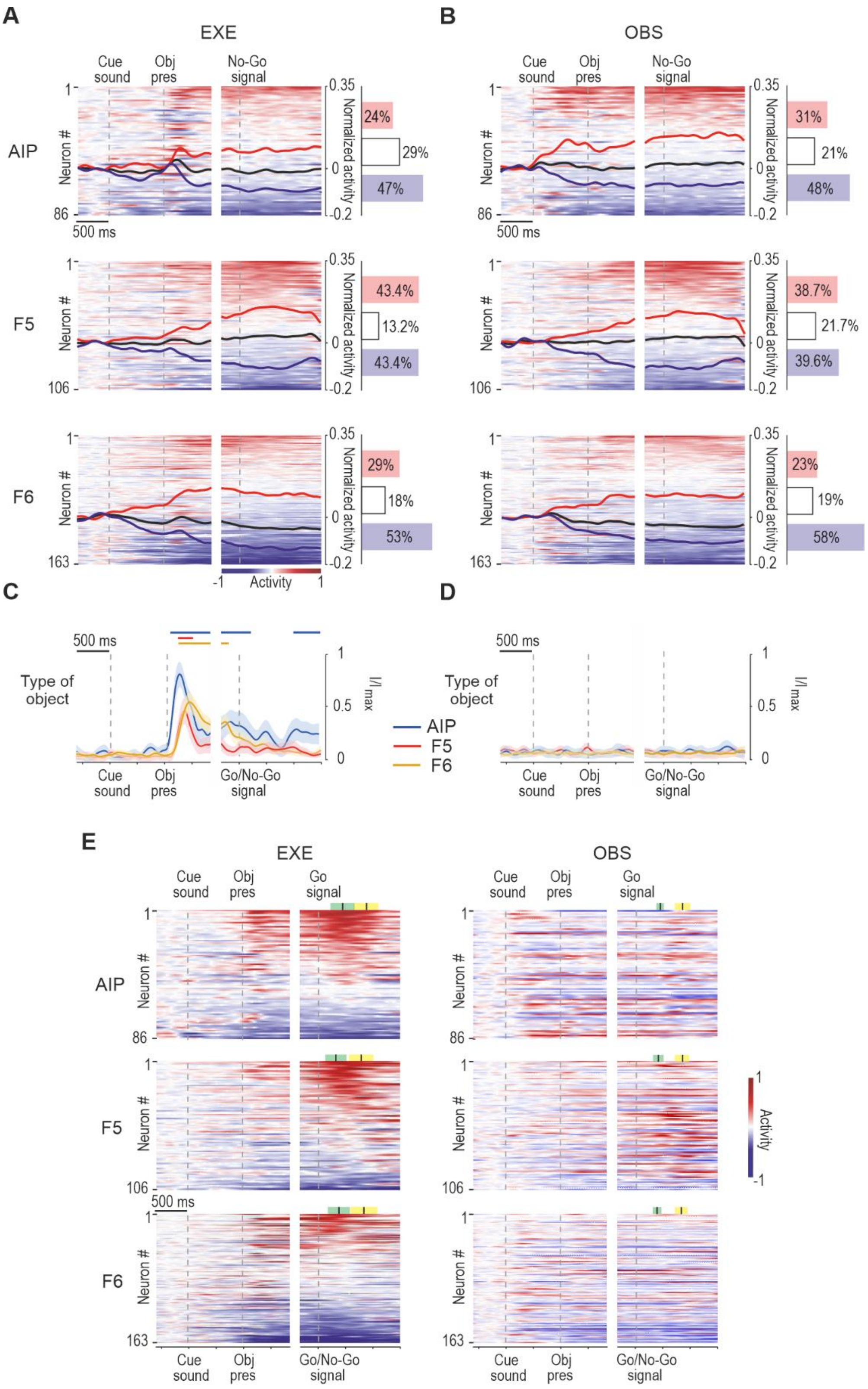

Figure S1. Functional properties of AIP, F5 and F6 neurons during No-Go trials of EXE and OBS. Related to Figure 2.

(A) Heat maps of all the recorded neurons in each area during No-Go trials of EXE. Each line represents one cell, and cells are ordered (from top to bottom) based on the magnitude of their activity with respect to baseline (red = facilitated, blue = suppressed) in the interval between 300 ms before, until 900 ms after, the No-Go signal. Black lines represent the averaged response of each population as a whole. The histograms on the right

indicate the percentage of facilitated, suppressed and non-significant neurons in each area (see Materials and Methods).

(B) Heat maps and population response of all the recorded neurons in each area during OBS. All conventions as in (A). Note that the neurons have been ordered independently from panel (A).

(C) Mutual information on type of object in EXE No-Go trials decoded from neuronal population activity of each area along the task unfolding period. Continuous colored bars on top of each plot indicate the period in which the decoding accuracy is significantly higher than chance (z-test on real versus shuffled data, see Materials and Methods).

(D) Mutual information on type of object (bottom) of OBS. Conventions as in (C).

(E) Heat maps of all the recorded neurons in each area during EXE and OBS, using in both tasks the same arrangement of the neurons applied in EXE (for comparison see Figure 2).

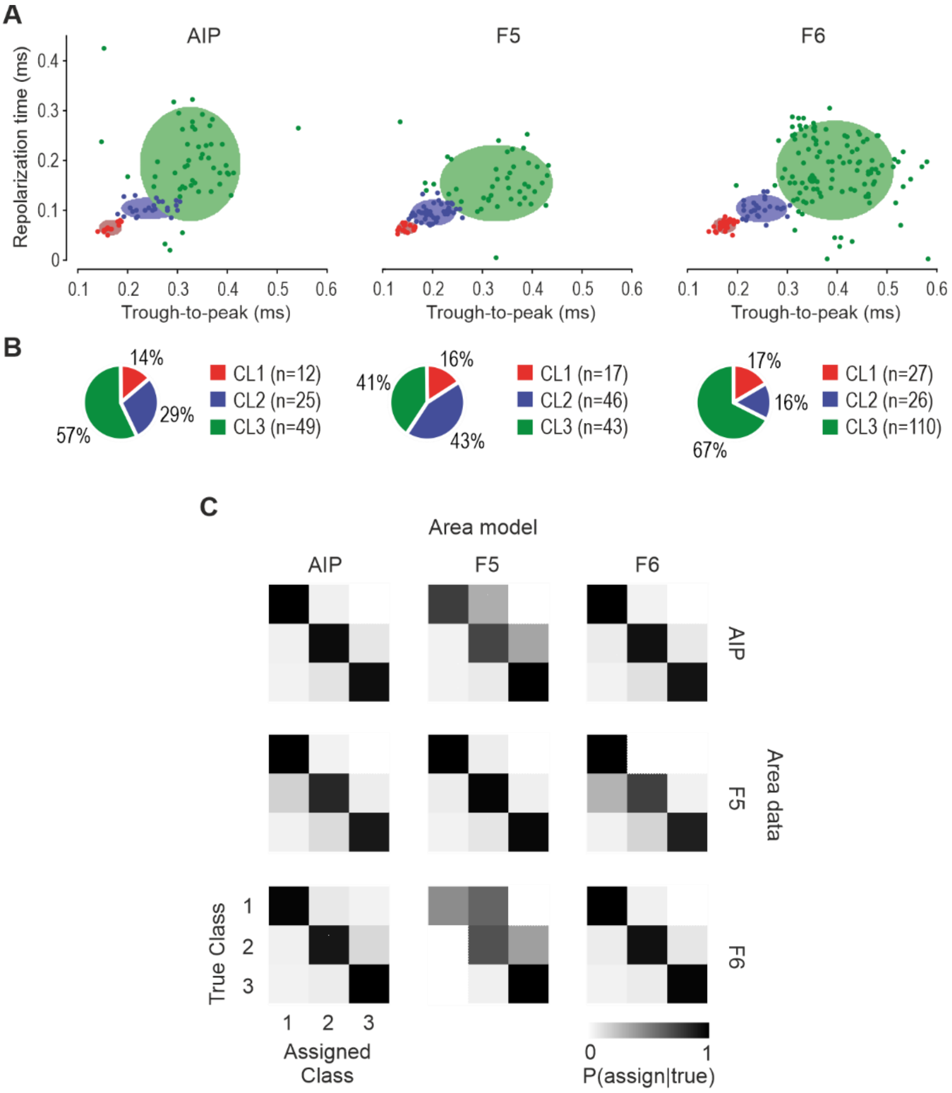

Figure S2. Reliability of waveform clustering within and across areas. Related to Figure 3.

(A) Projection of each spike waveforms in the 2D space formed by trough-to-peak duration and repolarization time: the clustering has been performed within each area, separately. Conventions as in main Figure 3A.

(B) Number of neurons in each cell class (in colour code) obtained from within-area clustering procedure illustrated in (A). Neurons in each class are expressed as a percentage of the total number of neurons recorded in that area. The distribution of neurons across cell classes is not significantly different from that obtained following clustering applied to the whole data set combined across areas (see Figure 5B) in AIP (χ^2 =0.71, p= 0.7) and F6 (χ^2=0.19, p=0.9), whereas it differs in area F5 (χ^2=18.27, p=0.0001), mostly because of a difference in the attribution of neurons to cell class 1 and 2.

(C) Estimation of cluster separation within each area and across areas. We extended the procedure used to estimate the cluster separation in the whole dataset (Figure 3B, and see STAR Methods) to assess whether and to what extent the clustering performed within individual areas generalizes to the other areas. For each pair of areas, 10^4 data points were randomly generated from the Gaussian mixture distribution of one area and assigned to classes based on the Gaussian mixture distribution of the other area. Thus, the diagonal plots of the resulting confusion matrix represent the cluster separation within each area, the off-diagonal plots represent how much the distribution of clusters is consistent across areas.

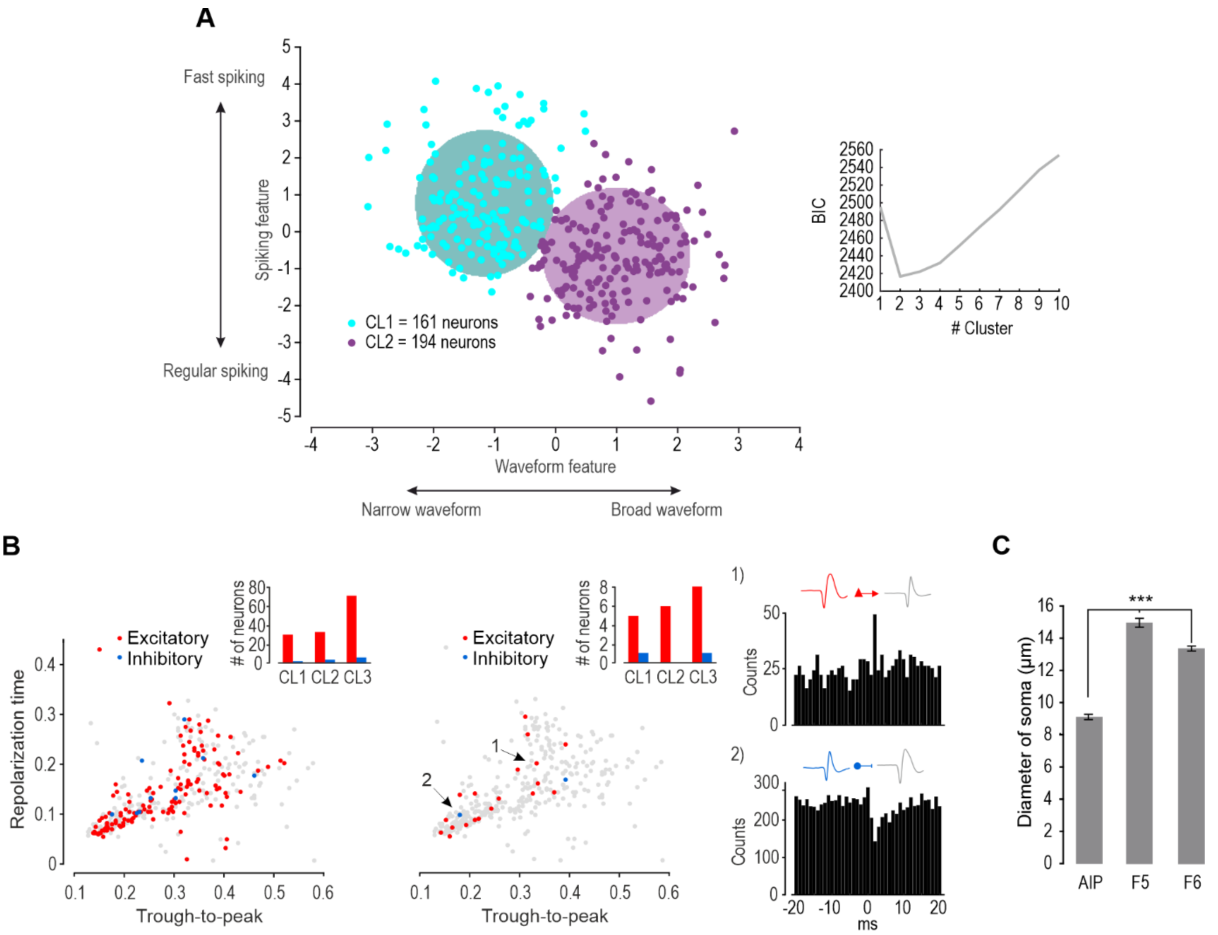

Figure S3. Clustering with spiking and waveform features, cross-correlograms and size of the cell soma in the three investigated areas. Related to Figure 3.

(A) Clustering of neurons based on combined waveform and spiking features. The clustering was performed by using a set of 6 dimensions: 3 spike shape features (Trough-to-peak, Repolarization time and Trough-amplitude ratio) and 3 neuronal firing features (Position of ISI's maximum, ISI's Coefficient of Variation, and Burst Index[S1]). We then reduced the dimensionality of the feature space by selecting a single "Waveform" and a single "Spiking" dimension by applying PCA within both set of features. Specifically, each feature was z-scored, PCA was performed within each set, and the projection onto the first principal component was selected as the score for the "Waveform" and "Spiking" features (the first PCs explained alone ~60% and ~70% of their total variance, respectively). Gaussian Mixture Model clustering was performed as in Figure 3A of the main text and Bayesian Information Criterion analysis revealed that the optimal number of clusters is 2.

(B) Cross-correlograms of all possible pairs of neurons. To investigate causal relationships among spikes of different neurons we calculated cross-correlogram histograms (CCHs) for all pairs of simultaneously recorded neurons with the criteria applied by Merchant et al. (2008)[S2]. Since significant 1 ms-lag suppression between neurons recorded from the same channel are likely an epiphenomenon of their close proximity[S3] we excluded CCHs between neurons recorded from the same channel. 5654 CCHs fulfilled the above criteria and were examined, and 314 of them were significant. Since some neurons appeared to trigger in an opposite way (excitatory vs inhibitory) multiple cells (n = 76 pairs including one of such neurons), they have been excluded. We obtained 238 significant pairs, including 143 distinct triggering neurons. Of them, 133 (red dots in left panel) were associated to excitatory effect whereas 10 (blue dots) to inhibitory effects. We then reasoned that the high number of excluded pairs because of unreliable, opposite (facilitatory vs inhibitory) effects exerted on different neurons makes plausible to consider that the criteria was too permissive. Thus, we also tried to apply more restrictive criteria, that is, 1) ± 4 SD threshold, 2) exclusion of all those pairs in which the CCH peak was higher than 1 SD from the second-highest bin in the 40 ms time window, 3) inhibition accepted as valid only when it lasted for at least two consecutive bins. Within the 24 pairs fulfilling these restrictive criteria, 21 different triggering neurons were identified (central panel). Among them, 19 showed excitatory interactions

and 2 showed inhibitory interactions (right panel). These findings suggest that in our data set triggering neurons with facilitatory effects can exhibit thin spikes and neurons with inhibitory effects can exhibit broad spikes.

(C) Each bar represents the average diameter of somata in each area (one-way ANOVA $F=977.65$, $p<0.001$). To measure each cell soma diameter, we used the measure function of the Nis-element software (Nikon Instruments Inc.). The histological material, prepared and processed as previously described[S4], was constituted by 3 sections digitized and photographed with a × 20 objective taken from the recorded regions of each area; from each section we sampled three 250 µm columns in order to have a uniform representation of cells from all the cortical layers. For each column, we plotted all the surface of the soma of each neuron for which the nucleus could be identified (AIP, n = 847; F5, n = 295; F6, n = 943).

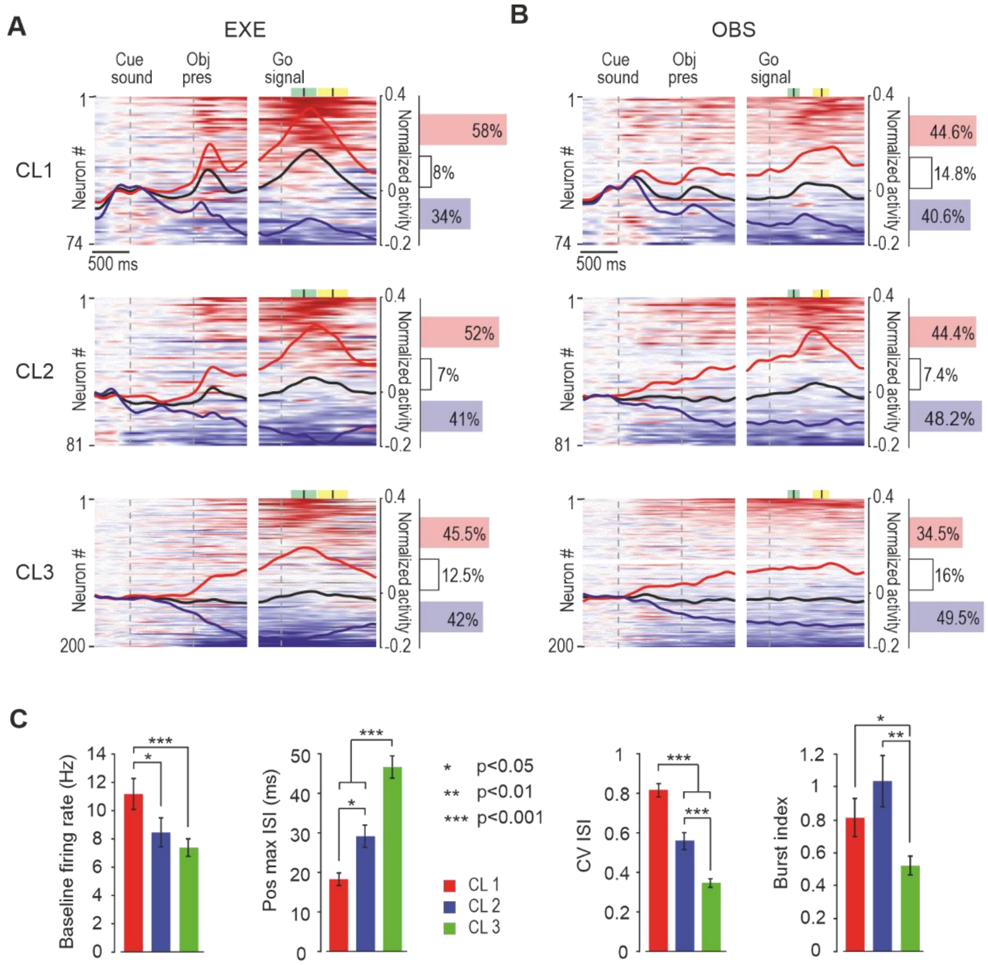

Figure S4. Tuning and firing properties of neurons in different cell classes. Related to Figure 4.

(A) Heat maps and population response of all the recorded neurons in each cell class during EXE. All conventions as in Figure S1A (all χ^2 comparisons between pairs of cell classes, p>0.11).

(B) Heat maps and population response of all the recorded neurons in each class during OBS. All conventions as in Figure S1A (all χ^2 comparisons between pairs of cell classes, p>0.12).

(C) From left to right: average baseline firing rate of each cell class during EXE (Mann-Whitney test); average position of the maximum of the ISI distribution (Mann-Whitney test); coefficient of variation of the ISI distribution (one-way ANOVA p<0.001, Tukey-Kramer post-hoc); average Burst index, calculated as the ratio of ISI intervals < 5ms divided by all intervals < 100ms, normalized by the same ratio that would be expected by a Poisson process of equal mean rate (Mann-Whitney test [S1]). Error bars within each plot indicate standard errors.

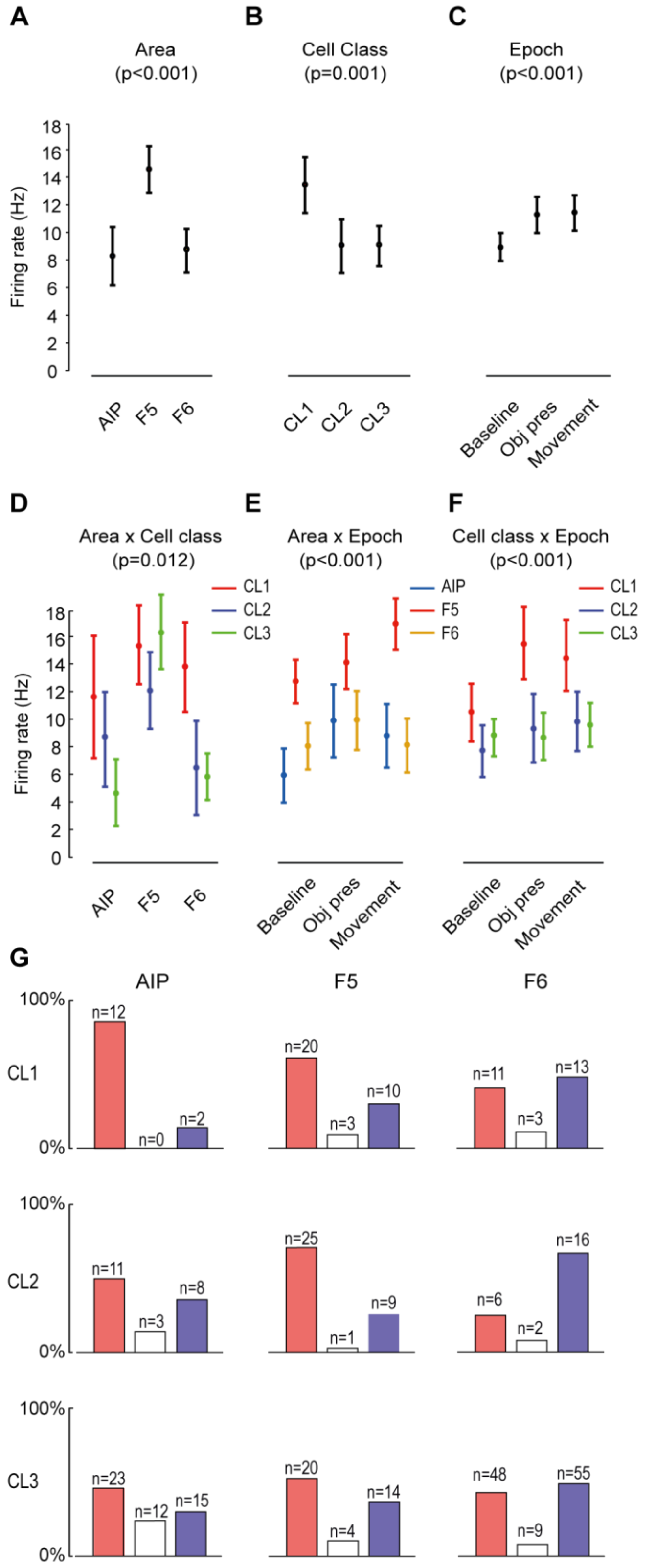

Figure S5. Tuning of different cell classes in different areas during EXE. Related to Figure 5.

(A) Main effect of Area (F=15.44, df=2, $p<0.001$) indicates that neurons of area F5 display greater firing rate than those of the other two areas (F5-AIP, $p<0.001$; F5-F6, $p<0.001$), which in turn did not differ from each other ($p=0.64$).

(B) Main effect of Cell class (F=6.75, df=2, $p=0.001$) indicates that neurons of cell class 1 have greater firing rate than those of the other two classes (CL1 vs CL2, $p<0.001$; CL1 vs CL3, $p<0.001$), which in turn did not differ from each other ($p=0.18$).

(C) Main effect of Epoch ($F=17.08$, $df=2$, $p<0.001$) indicates that, relative to baseline, the firing rate is greater during object presentation ($p=0.001$) and movement ($p<0.001$), which in turn did not differ from each other ($p=0.2$).

(D) Interaction between Cell class and Area ($F=3.26$, $df=4$, $p=0.012$) shows that class 1 neurons exhibit greater firing rate than those of the other two classes in F6 ($p<0.05$ for both comparisons) and of class 3 neurons in AIP ($p=0.026$). Furthermore, neurons in class 3 of F5 has a greater firing rate than class 3 neurons of the other areas ($p<0.001$ for both comparisons), which in turn did not differ from each other ($p=0.57$).

(E) Interaction between Area and Epoch ($F=8.58$, $df=4$, $p<0.001$) indicates that area F5 neurons discharge stronger than those of the other areas in all epochs, including baseline; their firing rate during movement is higher than during both baseline ($p<0.001$) and object presentation ($p<0.001$), which in turn did not differ from each other ($p=0.07$). AIP neurons significantly increase their firing rate relative to baseline during both object presentation epoch ($p=0.005$) and movement ($p=0.03$), which in turn did not differ from each other. Area F6 neurons did not show any overall modulation of their firing rate across epochs ($p>0.81$ for all comparisons).

(F) Interaction between Cell class and Epoch ($F=5.27$, $df=4$, $p<0.001$) indicates that during baseline epoch all cell classes exhibit similar firing rates ($p>0.1$ for all comparisons). In contrast, neurons of class 1 showed greater firing rate relative to baseline during object presentation and movement epochs ($p<0.001$ for both comparisons) and greater firing rate relative to neurons in cell class 2 and 3, in all epochs ($p<0.005$ for all comparisons).

(G) Percentage of facilitated (red), suppressed (blue) and non-significant (white) neurons within areas and cell classes in EXE.

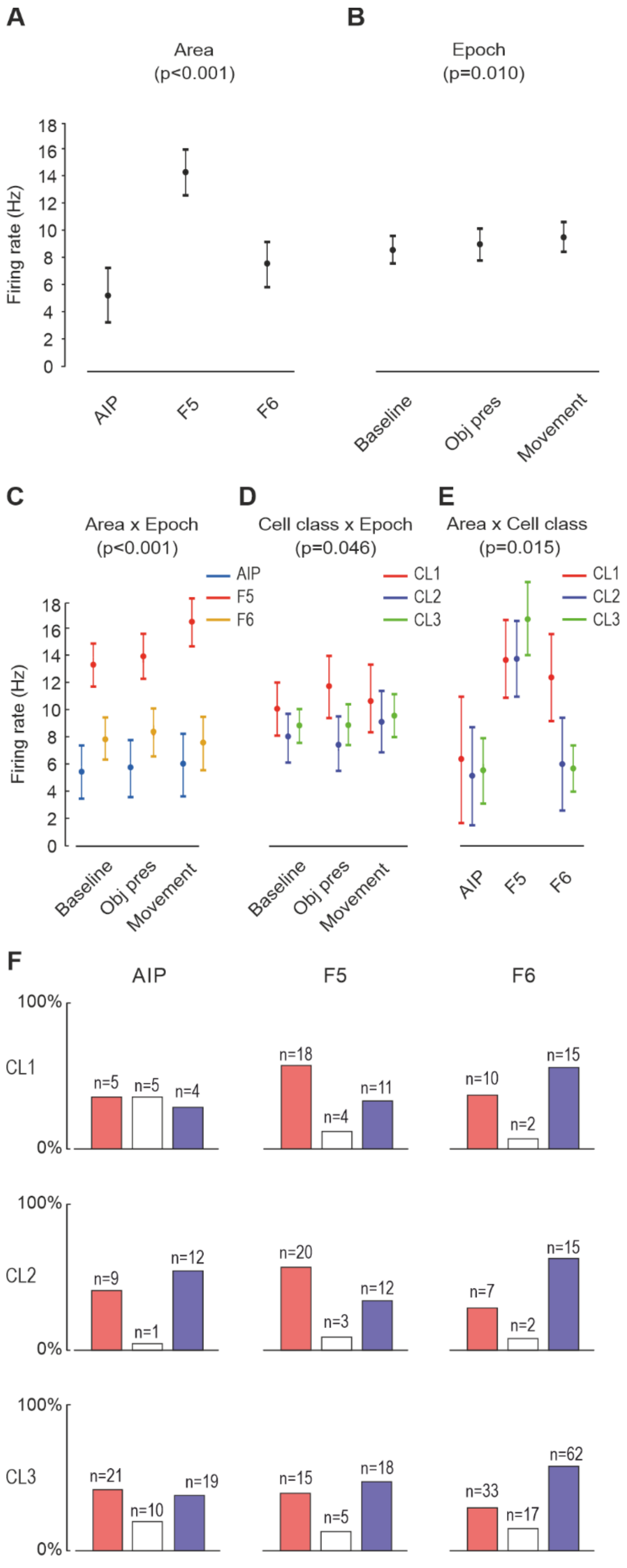

Figure S6. Tuning of different cell classes in different areas during OBS. Related to Figure 5.

(A) Main effect of Area (F=26.42, df=2, p<0.001) indicates that neurons of area F5 display greater firing rate than those of the other two areas (F5-AIP, p<0.001; F5-F6, p<0.001), which in turn did not differ from each other (p=0.28).

(B) Main effect of Epoch ($F=4.63$, $df=2$, $p=0.01$) indicates that the firing rate during movement epoch was greater than baseline ($p=0.007$) and object presentation epoch ($p=0.02$), which in turn did not differ from each other ($p=0.45$).

(C) Interaction between Area and Epoch ($F=8.56$, $df=4$, $p<0.001$) indicates that area F5 neurons discharge stronger than those of the other areas in all epochs, including baseline; their firing rate during movement is higher than during both baseline ($p<0.001$) and object presentation ($p<0.001$), which in turn did not differ from each other ($p=0.20$). Area F6 and AIP neurons did not show any overall modulation of their firing rate across epochs ($p>0.28$ for all comparisons).

(D) Interaction between Cell class and Epoch ($F=2.44$, $df=4$, $p=0.046$) indicates that during baseline all cell classes exhibit similar firing rates ($p>0.09$ for all comparisons). In contrast, neurons of class 1 showed greater firing rate relative to baseline during object presentation and movement epochs ($p<0.05$ for both comparisons), which in turn did not differ from each other ($p=0.27$), and neurons of class 2 showed greater firing rate during movement relative to baseline and object presentation ($p<0.005$), which in turn did not differ from each other ($p=0.67$).

(E) Interaction between Cell class and Area ($F=3.11$, $df=4$, $p=0.015$) shows that, in F6, class 1 neurons exhibit greater firing rate than those of the other two classes ($p<0.05$ for both comparisons), whereas in AIP and F5 no significant difference between cell classes emerge ($p>0.20$).

(F) Percentage of facilitated (red), suppressed (blue) and non-significant (white) neurons within areas and cell classes in OBS.

A

OBS \ EXE	CL1 F	CL1 S	CL1 NS	CL2 F	CL2 S	CL2 NS	CL3 F	CL3 S	CL3 NS
F	23	10	0	23	13	0	35	23	11
S	14	11	5	17	18	4	40	50	9
NS	6	4	1	2	2	2	16	11	5

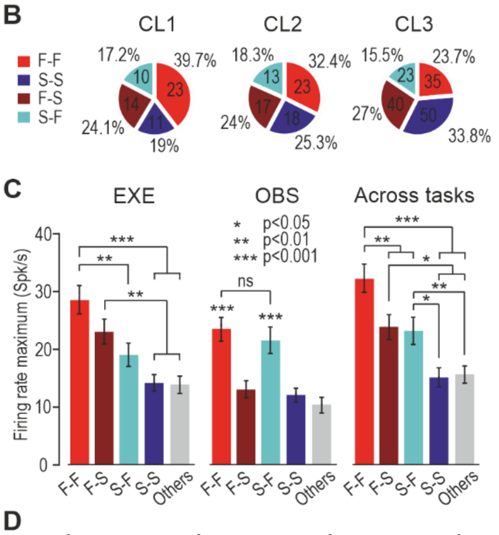

D

	AIP CL1	AIP CL2	AIP CL3	F5 CL1	F5 CL2	F5 CL3	F6 CL1	F6 CL2	F6 CL3	TOT
FF	4	4	7	12	12	5	2	3	9	58
SS	0	3	3	2	2	6	3	7	14	40
TOT AREA	21 (24.4%)			39 (36.8%)			38 (23.3%)			

E

	AIP FF	AIP SS	AIP OTs	F5 FF	F5 SS	F5 OTs	F6 FF	F6 SS	F6 OTs	TOT
Mk1	/	/	/	17	7	28	4	10	20	86
Mk2	3	5	10	19	9	26	19	42	68	201
Mk3	19	6	43	/	/	/	/	/	/	68
TOT AREA	22	11	53	36	16	54	23	52	88	

Figure S7. Distribution and properties of neurons in each class depending on their modulation in EXE and OBS. Related to Figure 6.

(A) Number of neurons with facilitated (F), suppressed (S) or non-significant (NS) response during EXE and OBS, across cell classes. Note that unselective neurons in both EXE and OBS are n = 8.

(B) Number of F-F, S-S, F-S and S-F neurons across cell classes (the first letter refers to EXE, the second one to OBS). Percentages are relative to the sum of these four paired classes.

(C) Firing rate maxima across paired classes. Maxima were obtained by taking the firing rate (trial- and object-averaged and smoothed with a 60 ms Gaussian kernel) of each neuron aligned to the object presentation (in the interval -1.3/0.7 s relative to this event) and Go/No-Go signal (in the interval -0.3/1.2 s relative to this event). The absolute maximum in this entire recording period within or across EXE and OBS is shown.

(D) Number of neurons with average MMD > 0 across paired classed, cell classes and areas. The average was taken within the movement epoch (0/0.7 s after movement onset).

(E) Number of F-F, S-S and Others neurons subdivided in areas and animals they were recorded from.

Supplemental References

S1. Constantinidis, C., and Goldman-Rakic, P.S. (2002). Correlated Discharges Among Putative Pyramidal Neurons and Interneurons in the Primate Prefrontal Cortex. J. Neurophysiol. *88*, 3487–3497.

S2. Merchant, H., Naselaris, T., and Georgopoulos, A.P. (2008). Dynamic Sculpting of Directional Tuning in the Primate Motor Cortex during Three-Dimensional Reaching. J. Neurosci. *28*, 9164 LP – 9172.

S3. Barthó, P., Hirase, H., Monconduit, L., Zugaro, M., Harris, K.D., and Buzsáki, G. (2004). Characterization of Neocortical Principal Cells and Interneurons by Network Interactions and Extracellular Features. J. Neurophysiol. *92*, 600–608.

S4. Albertini, D., Gerbella, M., Lanzilotto, M., Livi, A., Maranesi, M., Ferroni, C.G., and Bonini, L. (2020). Connectional gradients underlie functional transitions in monkey pre-supplementary motor area. Prog. Neurobiol. *184*, 101699.
